# Re-examining the Role of Coping Strategies in the Associations Between Infertility-Related Stress Dimensions and State-Anxiety: Implications for Clinical Interventions With Infertile Couples

**DOI:** 10.3389/fpsyg.2020.614887

**Published:** 2020-12-22

**Authors:** Maria Clelia Zurlo, Maria Francesca Cattaneo Della Volta, Federica Vallone

**Affiliations:** ^1^Dynamic Psychology Laboratory, Department of Political Science, University of Naples Federico II, Naples, Italy; ^2^Department of Humanities, University of Naples Federico II, Naples, Italy

**Keywords:** coping strategies, gender, infertility-related stress, moderating role, state-anxiety

## Abstract

Research has shown a direct relationship between infertility-related stress and anxiety in infertile patients. The present study goes into this relationship in depth, testing the moderating role of coping strategies (Seeking Social Support, Avoidant, Positive Attitude, Problem-Solving, Turning to Religion) in the associations between specific infertility-related stress dimensions (Social Concern, Need for Parenthood, Rejection of Childfree Lifestyle, Couple’s Relationship Concern) and State-Anxiety among male and female partners of infertile couples. Gender differences were also explored. Both members of 254 infertile couples completed a questionnaire consisting of Socio-demographics, Fertility Problem Inventory–Short Form (FPI-SF), Coping Orientation to Problem Experienced–New Italian Version (COPE-NIV), and State-Trait Anxiety Inventory-Y (STAI-Y). The results revealed that Social Concern and Couple’s Relationship Concern, in both partners, and Need for Parenthood, in female partners, had positive correlations with State-Anxiety. Seeking Social Support and Avoidant coping were related to increasing levels of State-Anxiety in both partners, whereas Positive Attitude coping strategies were related to lower levels of State-Anxiety in female partners. Problem-Solving and Avoidant coping played moderating roles between specific infertility-related stress dimensions and State-Anxiety in unexpected directions. Problem-Solving exacerbated the negative effects of Social Concern, whereas Avoidant coping buffered the negative effects of several infertility-related stress dimensions in both partners. Interventions to improve stress management and psychological health in infertile couples should consider that the adequacy of coping strategies is inherently situation specific. It therefore follows that patient-centered clinical interventions should consider the potential inadequacy of promoting Problem-Solving strategies, and that even Avoidance can be an efficient strategy for dealing with specific infertility-related stress dimensions.

## Introduction

Infertility condition was recognized among the greater stressors that may occur in life (Maroufizadeh et al., 2019). It, indeed, may expose infertile individuals and couples to an unexpected life crisis, characterized by loss of self-esteem, perception of disruption in the developmental trajectory of adulthood, inability to plan future, changes in identity and worldviews, and in personal, dyadic, and social relationships (Wischmann and Kentenich, 2017; Rooney and Domar, 2018; Shreffler et al., 2020; Sormunen et al., 2020).

In line with this, a large body of research demonstrated that high levels of stress and anxiety symptoms are frequently occurring psychological disorders among infertile patients (Mori et al., 1997; Chen et al., 2004; Verhaak et al., 2005; Dancet et al., 2010; Turner et al., 2013; De Berardis et al., 2014; Pawar et al., 2019; Kiani et al., 2020; Yazdi et al., 2020). Although anxiety is a normal adaptive response of individuals in stressful situations (Semple and Smyth, 2019), research performed in international context underlined that the prevalence of anxiety in members of infertile couples is significantly higher than in fertile controls and in the general population (Anderson et al., 2003; Matthiesen et al., 2011; Fallahzadeh et al., 2019; Kiani et al., 2020). Therefore, because both the prevalence and incidence of stress and anxiety symptoms stemming from infertility condition are worthy of note, research efforts were made to develop studies targeting a greater understanding of infertility-related stress process.

In this direction, in the last decades, two main traditions of research were developed. In particular, one branch of research explored the impact of Assisted Reproductive Technology (ART) treatments on quality of life and psychological health reported by infertile couples with the aim of improving service delivery and supporting infertile couples in dealing with medical treatments (Verhaak et al., 2005; Boivin et al., 2012; Gameiro et al., 2015; Agostini et al., 2017). Indeed, beyond the significant physical burden, ART treatment-related experiences may elicit adverse emotional outcomes linked to the uncertainty of the pregnancy achievement as well as feeling of hopelessness after treatment failures (Verhaak et al., 2005). Moreover, several studies also highlighted that intense and protracted experiences of stress and psychological disease may also have a significant impact on ART treatment success, including follow-ups (Smeenk et al., 2001; Gürhan et al., 2009; Turner et al., 2013; Vellani et al., 2013; Purewal et al., 2018), potentially resulting in a vicious circle.

The other branch of research recognized infertility experience in itself as a potential hindrance to psychological health of infertile patients at individual and couple levels (Newton et al., 1999). From this perspective, indeed, given the distinct feature of infertility experience, research has identified specific infertility-related stress dimensions characterizing infertility condition, namely, perceived social concerns (i.e., feelings of isolation; perceived alienation; discomfort and stress in spending time with family and/or peers; sensitivity to comments and reminders of infertility), concerns related to need for parenthood (i.e., parenthood as essential step to achieve own identity, and as fundamental life goal), concerns related to rejection of a future without a child (i.e., negative view of a childfree lifestyle; satisfaction and/or happiness as dependent on achieving parenthood), and, finally, concerns about the impact of infertility on the couple relationship (i.e., difficulty in talking about infertility with the partner; reduced intimacy and sexual enjoyment; diminished self-esteem) (Newton et al., 1999; Zurlo et al., 2017). These specific infertility-related stress dimensions were widely demonstrated to be significant predictors of infertile patients’ psychological disease (Lakatos et al., 2017; Pozza et al., 2019). This fostered the development of further research aiming at identifying protective factors potentially reducing perceived stress and psychological disease among infertile couples (Donkor and Sandall, 2007; Sreshthaputra et al., 2008).

In this research direction, following the transactional approach underpinning stress-coping models on adjustment to chronic stressors (Lazarus and Folkman, 1984), research explored the effects of the interplay between individual characteristics (e.g., personality characteristics and coping strategies) and situational characteristics (e.g., infertility-related stress dimensions and parameters) in influencing infertility-related stress process and psychological health conditions in infertile patients (Van den Broeck et al., 2010; Zurlo et al., 2018, 2020).

In particular, because understanding the role of coping strategies is considered pivotal in explaining individual differences in emotional response to infertility-related stress dimensions as well as to develop preventive tailored interventions (Verhaak et al., 2007), a large body of research investigated their role in influencing infertile patients’ perceived stress and psychological well-being; however, this produced contrasting and mixed evidence.

Specifically, several studies supported the protective role of positive attitude/reinterpretation (Berghuis and Stanton, 2002; Benyamini et al., 2008; Gourounti et al., 2012), seeking social support (Schmidt et al., 2005; Rashidi et al., 2011; Faramarzi et al., 2013), and problem-solving coping strategies (Berghuis and Stanton, 2002; Gourounti et al., 2012; Faramarzi et al., 2013), as well as the detrimental effect of escape/avoidance coping (Schmidt et al., 2005; Peterson et al., 2006; Lykeridou et al., 2011; Gourounti et al., 2012; Faramarzi et al., 2013).

Notwithstanding, a growing branch of research underlined the view that avoidant strategies are not limited to denial and disengagement, since including strategies such as positive distraction (Kleiber et al., 2002; Waugh et al., 2020). It was, therefore, emphasized that the recourse to positive distraction (e.g., thinking about and/or engage in other activities) may disclose the possibility to distance oneself from goals being threatened by the stressor, so inducing positive emotions. In line with this, a recent study revealed that active-distractive coping was significantly associated with lower levels of psychological disease in infertile women (Khalid and Dawood, 2020).

In the same direction, some studies also found no evidence supporting neither the expected negative role of avoidant coping nor the protective role of problem-focused strategies among infertile patients (Verhaak et al., 2005), highlighting that planning and seeking social support coping strategies could even be associated with infertile patients’ impaired psychological well-being (Benyamini et al., 2008).

Finally, mixed evidence also emerged concerning the adoption by infertile patients of coping strategies centered on religious and spiritual beliefs, which revealed both negative (Berghuis and Stanton, 2002; Oti-Boadi and Asante, 2017) and protective effects (Benyamini et al., 2008; Latifnejad Roudsari et al., 2014; Casu et al., 2018).

Notwithstanding the mixed and contrastive literature, the detrimental impact of infertility-related stress dimensions on anxiety and the meaningful direct contribution of coping strategies in influencing infertility-related stress process are well demonstrated. However, further research is needed to clarify the possible interplay and complex pathways of associations between infertility-related stress dimensions, coping strategies, and perceived levels of psychological disease in terms of anxious symptoms.

In addition, although research increasingly emphasizes that infertility condition may have a significant impact on both partners of infertile couples, some gender differences were also reported (e.g., Berghuis and Stanton, 2002; Ying et al., 2015; Molgora et al., 2019). However, whether the majority of studies underlined that women perceive higher levels of infertility-related stress (Cserepes et al., 2013; Luk and Loke, 2015) and anxiety (El Kissi et al., 2013; Ying et al., 2015; Schaller et al., 2016), mixed evidence on gender differences in coping strategies were found. Indeed, on the one side, some studies highlighted that infertile women were more likely to recur to seeking social support and escape/avoidance when compared with men, whereas men used greater amounts of self-controlling (Mohammadi et al., 2018) and planful problem-solving (Peterson et al., 2006), while, on the one other side, a review conducted by Jordan and Revenson (1999) highlighted that women display higher adoption not only of seeking social support and escape/avoidance but also of plan-oriented problem-solving and positive reappraisal.

Consequently, considering all the research reported previously, there is increasing interest to achieve a greater understanding of infertility-related stress and coping processes, also taking into account potential gender differences.

Therefore, the present study aims to focus on the associations of infertility-related stress dimensions (Social Concern, Need for Parenthood, Rejection of Childfree Lifestyle, Couple’s Relationship Concern) with State-Anxiety reported by male and female partners of infertile couples, exploring gender differences and evaluating the potential specific moderating role of adopted Coping strategies (Seeking Social Support, Avoidant, Positive Attitude, Problem Solving, Turning to Religion). Indeed, because of the necessity to actively counteract and prevent the detrimental effects of protracted high levels of stress and anxiety among infertile patients, this approach would allow gaining further evidence-based information to develop tailored patient-centered counseling interventions (Lorah and Wong, 2018; Liw and Han, 2020).

In line with the aim of the present study, the research hypotheses are as follows:

H1. Women perceive higher levels of infertility-related stress and state-anxiety than men. No hypotheses were made about gender differences in coping strategies due to the mixed evidence reported in the literature.

H2. Infertility-related stress dimensions are significantly and positively related to state-anxiety in male and female partners of infertile couples.

H3. Coping strategies are significantly related to state-anxiety in male and female partners of infertile couples. No prediction was made about the direction of the relationships due to the mixed evidence reported in the literature.

H4. Coping strategies moderate the relationships between infertility-related stress dimensions and state-anxiety in male and female partners of infertile couples.

## Materials and Methods

### Participants and Sampling

This cross-sectional multi-center study was conducted between September 2017 and September 2019 in 10 Italian centers of assisted reproduction of Brescia, Naples, and Udine. Participants were 254 couples (254 male, 254 female) undergoing ART treatments. Chairpersons were contacted to consent the authorization for administering a questionnaire in their centers and, after obtaining their adhesion to the project, infertile couples were directly asked to participate in the study before their medical appointment. All infertile patients were fully informed about the purpose of the current study. They were assured about the confidentiality of the data, and they were informed that the data would be used only for the aim of the research and refusal to participate would not influence their current and future treatments in any way. The current study is part of a larger project on factors influencing psychological well-being of infertile couples, and therefore, the study dataset partially overlaps with those used in a previous study (Zurlo et al., 2020). The project was approved by the Ethical Committee of Psychological Research of the University of Naples Federico II (IRB:34/2019). Research was performed in accordance with the 1964 Helsinki declaration and its later amendments or comparable ethical standards. Every precaution was taken to protect the privacy of participants and the confidentiality of their personal information, and the questionnaires were completed anonymously. Informed consent was obtained from each patient before participating in the study. In total, 350 couples were asked to individually complete a questionnaire lasting 20–25 min (one session) in a quiet room setting in the medical center, and one of the authors was present to answer any queries raised by participants. If one or both members of infertile couples refused to complete the questionnaire they were not included in the final dataset. Overall, 254 couples (254 male, 254 female) completed the questionnaire (response rate: 72.57%). All the couples included were diagnosed with primary infertility.

### Measures

#### Background Information

The questionnaire included a section dealing with background information, containing questions on socio-demographic characteristics, i.e., Gender, Age (in years), Educational Level (Upper Secondary School/College), and Employment status (Unemployed/Employed), and on infertility-related parameters, i.e., Duration of infertility (in years), Type of Diagnosis (Female Factor, Male Factor, Combined Factor, and Unexplained Factor), and presence of Previous Treatments (No/Yes).

#### Infertility-Related Stress Dimensions

Infertility-related stress dimensions were measured by using the Fertility Problem Inventory–Short Form (FPI-SF; Italian version: Zurlo et al., 2017), which consists of 27 items on a six-point Likert scale ranging from one (Strongly disagree) to six (Strongly agree) divided into four subscales: Social Concern (10 items; Cronbach’s α = 0.88); Need for Parenthood (six items; Cronbach’s α = 0.88); Couple’s Relationship Concern (five items; Cronbach’s α = 0.70); Rejection of Childfree Lifestyle (six items; Cronbach’s α = 0.77).

#### Coping Strategies

Coping strategies were measured by using the Coping Orientation to Problem Experienced–New Italian Version (COPE-NIV; Carver et al., 1989; Italian version: Sica et al., 2008), which consists of 60 items on a five-point Likert scale ranging from one (I usually don’t do this at all) to four (I usually do this a lot) divided into five subscales: Seeking Social Support (12 items; Cronbach’s α = 0.88); Avoidant (16 items; Cronbach’s α = 0.70); Positive Attitude (12 items; Cronbach’s α = 0.76); Problem Solving (12 items; Cronbach’s α = 0.83); Turning to Religion (8 items; Cronbach’s α = 0.85).

#### State-Anxiety

Anxiety symptoms were measured by using the State scale from the State-Trait Anxiety Inventory (STAI-Y; Spielberger, 1972; Italian version: Pedrabissi and Santinello, 1989), which consists of 20 items on a four-point Likert scale ranging from one (Not at all) to four (Very much). Total score ranges from 20 to 80 (Cronbach’s α = 0.91). State-Anxiety scores were also converted into percentages and, according to the Italian validation study (Pedrabissi and Santinello, 1989), a score of 50.93 for female partners and 45.70 for male partners were considered to be the cut-off point to define the clinical cases.

### Data Analysis

The SPSS statistical program (version 21) was used to perform descriptive analyses and correlation analysis. First, descriptive statistics were conducted according to gender. Therefore, to address hypothesis 1 on gender differences in study variables (H1), *t*-tests were carried out to compare mean scores of infertility-related stress dimensions, coping strategies, and State-Anxiety according to gender. Second, Pearson’s correlations between the study variables were undertaken for the two genders to test, respectively, the hypothesized correlations between infertility-related stress dimensions and State-Anxiety (H2), and between coping strategies and State-Anxiety (H3). Finally, the Structural Equation Modeling (SEM) unconstrained approach put forward by Marsh et al. (2004) was carried out using AMOS (version 26) to test the hypothesized moderating role of coping strategies on the relationships between infertility-related stress dimensions and State-Anxiety in male and female partners of infertile couples, separately (H4).

## Results

### Characteristics of Participants

Individual characteristics and infertility-related parameters of study participants are illustrated in Table 1.

**TABLE 1:** Characteristics of study participants (*N* = 254 couples).

Variable	Female	Male	Couple
**Socio-demographic characteristics**			
Age [M ± SD (range)]	33.71 ± 3.66 (22–42)	35.60 ± 3.79 (24–48)	
Educational level [*N* (%)]			
Upper secondary school	51 (20.1%)	42 (16.5%)	
College	203 (79.9%)	212 (83.5%)	
Employment status [*N* (%)]			
Unemployed	63 (24.8%)	17 (6.7%)	
Employed	191 (75.2%)	237 (93.3%)	
**Infertility-related parameters**			
Duration of infertility [M ± SD (range)]			3.27 ± 2.64 (1–19)
Type of diagnosis [*N* (%)]			
Male factor			73 (28.7%)
Female factor			81 (31.9%)
Combined factor			61 (24.0%)
Unexplained			39 (15.4%)
Previous treatments [*N* (%)]			
No			107 (42.1%)
Yes			147 (57.9%)

The means and SDs of study variables for the two genders are summarized in Table 2. With respect to gender differences (H1), and, in particular, considering perceived levels of infertility-related stress dimensions, data revealed that women reported significantly higher levels of Social Concern (*t* = 1.98; *p* = 0.049), Need for Parenthood (*t* = 2.83, *p* = 0.005), and Couple’s Relationship Concern (*t* = 3.53, *p* < 0.001). There was no significant gender difference in perceived levels of Rejection of Childfree Lifestyle (*t* = 0.71, *p* = 0.476). With respect to coping strategies, women and men showed a similar recourse to strategies centered on Avoidance (*t* = 0.49, *p* = 0.622), Positive Attitude (*t* = 0.44, *p* = 0.660), Problem Solving (*t* = -0.50, *p* = 0.614), and Turning to Religion (*t* = 1.50, *p* = 0.133), whereas women reported greater recourse to Seeking Social Support coping (*t* = 3.85, *p* < 0.001).

**TABLE 2:** Means, SDs, and correlations between study variables for male and female partners of infertile couples.

	1	2	3	4	5	6	7	8	9	10	Mean	SD
1 Social Concern	1	0.09	0.13^∗^	0.43^∗∗^	–0.11	0.22^∗∗^	−0.43^∗∗^	−0.16^∗∗^	–0.06	0.52^∗∗^	26.26	12.08
2 Need for Parenthood	0.24^∗∗^	1	0.29^∗∗^	0.24^∗∗^	–0.01	−0.21^∗∗^	0.06	0.15^∗^	0.29^∗∗^	0.06	25.82	6.32
3 Rejection of Childfree Lifestyle	0.14^∗^	0.31^∗∗^	1	0.03	–0.03	−0.19^∗∗^	–0.08	0.03	0.33^∗∗^	–0.11	26.63	6.39
4 Couple’s Relationship Concern	0.44^∗∗^	0.32^∗∗^	0.03	1	0.20^∗∗^	0.32^∗∗^	–0.04	0.04	0.07	0.43^∗∗^	11.40	4.54
5 Seeking Social Support	–0.02	0.01	−0.14^∗^	0.19^∗∗^	1	0.50^∗∗^	0.60^∗∗^	0.32^∗∗^	0.16^∗^	0.27^∗∗^	24.27	7.10
6 Avoidant	0.11	−0.20^∗∗^	−0.17^∗∗^	0.18^∗∗^	0.36^∗∗^	1	0.25^∗∗^	0.16^∗∗^	0.00	0.57^∗∗^	25.12	7.71
7 Positive Attitude	−0.54^∗∗^	–0.11	−0.13^∗^	–0.10	0.44^∗∗^	0.28^∗∗^	1	0.46^∗∗^	0.17^∗∗^	–0.08	27.59	6.72
8 Problem Solving	–0.10	0.11	–0.03	0.07	0.36^∗∗^	0.24^∗∗^	0.35^∗∗^	1	0.01	0.07	28.82	6.78
9 Turning to Religion	0.05	0.31^∗∗^	0.16^∗^	0.20^∗∗^	0.25^∗∗^	0.02	0.17^∗∗^	0.21^∗∗^	1	–0.01	23.35	4.34
10 State-Anxiety	0.41^∗∗^	0.16^∗∗^	–0.06	0.32^∗∗^	0.20^∗∗^	0.39^∗∗^	−0.17^∗∗^	0.08	0.11	1	41.81	10.36
Mean	28.35	27.37	27.03	12.93	26.72	25.45	27.85	28.53	23.93	44.24		
SD	11.82	5.98	6.53	5.17	7.20	7.58	6.58	6.23	4.45	10.35		

Male partners’ results are reported above the diagonal. Female partners’ results are reported below. *p < 0.05. **p < 0.01.

Considering psychological health conditions, women reported significantly higher levels of State-Anxiety (*t* = 2.64, *p* = 0.008). Moreover, according to the clinical cut-off scores for State-Anxiety (i.e., scores ≥ 50.93 for women and ≥ 45.70 for men; STAI-Y; Pedrabissi and Santinello, 1989) it emerged that, respectively, 26.8% (*n* = 68) of female and 34.6% (*n* = 88) of male partners scored at clinical threshold for State-Anxiety. Overall, findings confirmed H1.

### The Correlation Analysis

Table 2 also displayed the intercorrelations of study variables for the two genders.

Concerning the correlations between Infertility-related stress dimensions and State-Anxiety (H2), Social Concern (men *r* = 0.52, *p* < 0.01; women *r* = 0.41, *p* < 0.01) and Couple’s Relationship Concern (men *r* = 0.43, *p* < 0.01; women *r* = 0.32, *p* < 0.01) were significantly and positively correlated with State-Anxiety in both partners, whereas Need for Parenthood was positively correlated with State-Anxiety in female partners only (*r* = 0.16, *p* < 0.01). No evidence supported significant correlations of Rejection of Childfree Lifestyle with State-Anxiety in both partners. Overall, findings partially confirmed H2.

Concerning the correlations between Coping strategies and State-Anxiety (H3), Seeking Social Support (men *r* = 0.27, *p* < 0.01; women *r* = 0.20, *p* < 0.01) and Avoidant coping strategies (men *r* = 0.57, *p* < 0.01; women *r* = 0.39, *p* < 0.01) were significantly and positively correlated to State-Anxiety in both partners, while Positive Attitude negatively correlated to State-Anxiety in female partners only (*r* = -0.17, *p* < 0.01). No evidence supported significant correlations of Problem Solving and Turning to Religion. Overall, findings partially confirmed H3.

### Moderating Effects

Infertility-related stress dimensions and coping strategies were entered into moderating models by using SEM. Data highlighted the significant moderating role of Problem Solving and Avoidant coping strategies, partially supporting H4.

**FIGURE 1:**
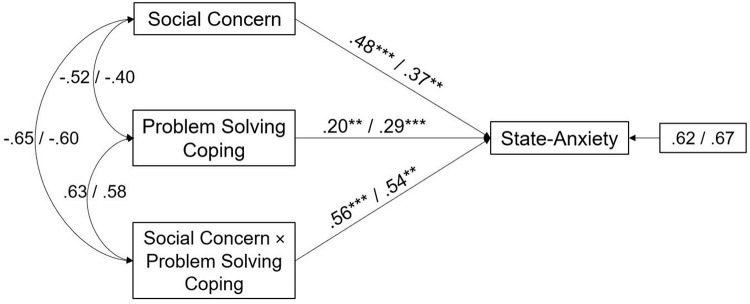
A moderate model of Social Concern and State-Anxiety through Problem Solving coping in male and female partners of infertile couples. Standardized regression coefficients are provided along the paths. The first coefficient in each path refers to men, whereas the second refers to women. ^∗∗^*p* < 0.01, ^∗∗∗^*p* < 0.001.

In particular, the interaction effect of Problem Solving coping and Social Concern was significant in both male and female partners (path analyses are shown in Figure 1). The main effect estimates for Problem Solving coping were, respectively, 0.20, *p* < 0.01 for male and 0.29, *p* < 0.001 for female partners, and the interaction effects were 0.56, *p* < 0.001 for male and 0.54, *p* < 0.01 for female. This suggests that Problem Solving coping significantly increased the negative effects of Social Concern on State-Anxiety in both partners.

**FIGURE 2:**
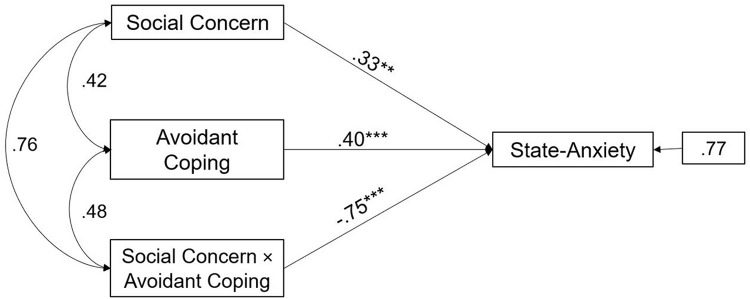
A moderate model of Social Concern and State-Anxiety through Avoidant coping in male partners of infertile couples. Standardized regression coefficients are provided along the paths. ^∗∗^*p* < 0.01, ^∗∗∗^*p* < 0.001.

Moreover, the interaction effect of Avoidant coping and Social Concern was significant in male partners (path analysis is shown in Figure 2). The main effect estimates for Avoidant coping were 0.40, *p* < 0.001 and the interaction effect was -0.75, *p* < 0.001. This suggests that Avoidant coping significantly buffered the negative effects of Social Concern on State-Anxiety in male partners.

**FIGURE 3:**
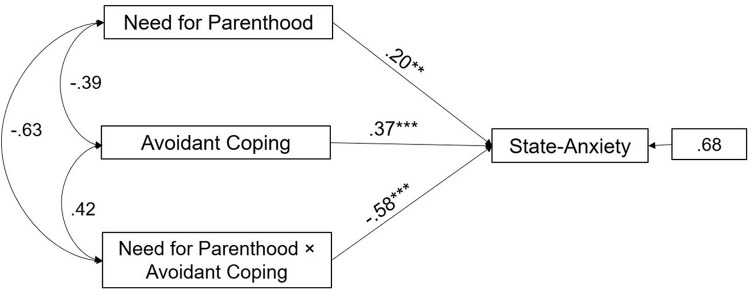
A moderate model of Need for Parenthood and State-Anxiety through Avoidant coping in female partners of infertile couples. Standardized regression coefficients are provided along the paths. ^∗∗^*p* < 0.01, ^∗∗∗^*p* < 0.001.

Likewise, the interaction effect of Avoidant coping and Need for Parenthood was significant in female partners (path analysis is shown in Figure 3). The main effect estimates for Avoidant coping were 0.37, *p* < 0.001 and the interaction effect was -0.58, *p* < 0.001. This suggests that Avoidant coping significantly buffered the negative effects of Need for Parenthood on State-Anxiety in female partners.

**FIGURE 4:**
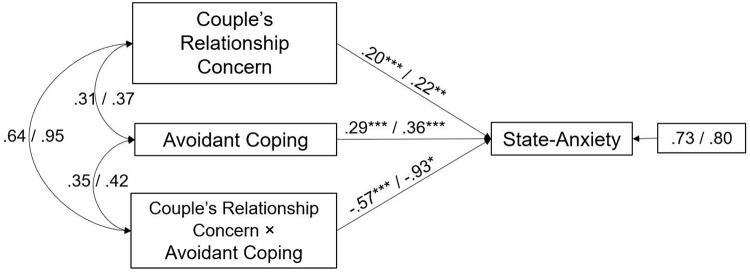
A moderate model of Couples’ Relationship Concern and State-Anxiety through Avoidant coping in male and female partners of infertile couples. Standardized regression coefficients are provided along the paths. The first coefficient in each path refers to men, whereas the second refers to women. *p < 0.05, **p < 0.01, ***p < 0.001.

Finally, the interaction effect of Avoidant coping and Couple’s Relationship Concern was significant in both male and female partners (path analyses are shown in Figure 4). The main effect estimates for Avoidant coping were, respectively, 0.29, *p* < 0.001 for male and 0.36, *p* < 0.001 for female partners and the interaction effects were -0.57, *p* < 0.001 for male and -0.93, *p* < 0.05 for female. This suggests that Avoidant coping significantly buffered the negative effects of Couple’s Relationship Concern on State-Anxiety in both partners.

## Discussion

The study aimed to investigate the associations between infertility-related stress dimensions and State-Anxiety in male and female partners of infertile couples, testing the moderating role of coping strategies. Findings provided original knowledge in the field of infertility-related stress process research, offering practical implications to foster efficacy in counseling interventions.

First, considering gender differences (H1), in line with previous research (Cserepes et al., 2013; Luk and Loke, 2015; Ying et al., 2015), data revealed that women reported significantly higher perceived levels of stress. In particular, women reported significantly higher levels of stress related to Social Concern, Need for Parenthood, and Couple’s Relationship Concern. However, no significant gender differences in perceived levels of Rejection of Childfree Lifestyle were found.

With respect to coping strategies, in line with previous studies (Jordan and Revenson, 1999; Peterson et al., 2006; Mohammadi et al., 2018), women reported significantly greater recourse to Seeking Social Support. Nonetheless, no other statistically significant gender differences were supported, and our findings suggested that women and men showed a similar adoption of coping strategies centered on Avoidance, Positive Attitude, Problem Solving, and Turning to Religion.

Considering psychological health conditions, although our findings supported the majority of studies indicating higher perceived levels of anxiety in women in comparison with men (e.g., El Kissi et al., 2013; Schaller et al., 2016), it emerged that 34.6% of men and 26.8% of women met the cut-off for clinical levels of State-Anxiety. Therefore, although it is clear that infertile women could be at higher risk than their partners—because they are proven to be involved to a greater extent both at physical and emotional levels—these remarkable findings highlighted the compelling need to target both male and female partners of infertile couples for the development of timely supportive counseling interventions.

From this perspective, overall, data from the present study confirmed the international research (Anderson et al., 2003; Matthiesen et al., 2011; Kiani et al., 2020) that underlined significantly higher levels of clinical anxiety in infertile patients than the general population (i.e., 5.1% of the Italian general population suffering from clinical Anxiety; de Girolamo et al., 2006). These findings supported the interest to explore, within the present study, the dynamic relationship between perceived infertility-related stress dimensions, adopted coping strategies, and levels of anxiety in male and female partners of infertile couples.

In this direction, in line with previous research (Lakatos et al., 2017; Pozza et al., 2019), the correlation analysis revealed that both social concerns (i.e., perceived discomfort in spending time with family/friends; sensitivity to comments and reminders of infertility; feelings of isolation) and couple’s relationship concerns (i.e., difficulty in talking about infertility with the partner; reduced intimacy/enjoyment/self-esteem) were significantly associated to increased levels of anxious symptoms in both male and female partners. Furthermore, perceived need for parenthood (i.e., considering parenthood as a fundamental life goal) was significantly associated to increased levels of anxiety in infertile women. Conversely, our data failed to support significant correlations between the rejection of childfree lifestyle (i.e., negative view of a childfree lifestyle; satisfaction and/or happiness as dependent on achieving parenthood) and anxiety both in female and in male infertile partners. We can hypothesize that this latter result could be connected to the possible effects of changes in Western countries’ beliefs concerning the role of parenthood in the definition of individuals’ identity, lifestyle, and life satisfaction. Such significant changes could be, therefore, considered as a further potential resource to be accounted for counseling interventions fostering individual and couple adjustment to infertility experience.

Overall, these findings highlighted those specific infertility-related stress dimensions significantly associated with anxiety symptoms in infertile patients, partially confirming H2. In addition, some gender specificities were also suggested.

With respect to the correlations between Coping strategies and State-Anxiety (H3), findings highlighted significant associations of Seeking Social Support, Avoidant, and Positive Attitude coping strategies, partially confirming H3.

In particular, in line with Benyamini et al. (2008), but in contrast with the majority of previous research (Schmidt et al., 2005; Rashidi et al., 2011; Gourounti et al., 2012; Faramarzi et al., 2013), our data revealed that Seeking Social Support was positively related to State-Anxiety in both partners. From this perspective, we could wonder whether these findings could unveil a potential side effect of adopting strategies centered on reliance on others, which could result in a vicious circle exacerbating feeling of apprehension, frustration, nervousness, and anxiety. Moreover, also considering our findings on the higher recourse by women to Seeking Social Support coping strategy, we can affirm that the recourse to this strategy deserves a careful exploration within interventions with infertile women.

Furthermore, in line with research underlining the detrimental effect of escape/avoidance coping on infertile patients’ psychological health conditions (Peterson et al., 2006; Lykeridou et al., 2011; Gourounti et al., 2012; Faramarzi et al., 2013), we found that Avoidant coping was positively related to State-Anxiety in both partners.

Conversely, Positive Attitude coping emerged to be negatively related to State-Anxiety in female partners, and, therefore, our findings fully supported those studies underlining the protective role of coping strategies centered on positive reinterpretation (Benyamini et al., 2008; Gourounti et al., 2012). Indeed, it is remarkable that Positive Attitude emerged—despite among women only—as the sole coping strategy directly associated with lower levels of anxiety symptoms. This suggested that a better adjustment to infertility experience could be promoted, within interventions with infertile women, by fostering individual processes of positive re-appraisal and reinterpretation of own condition.

No evidence was found supporting neither positive (Berghuis and Stanton, 2002; Oti-Boadi and Asante, 2017) nor negative (Latifnejad Roudsari et al., 2014; Casu et al., 2018) associations between Turning to Religion and perceived levels of State-Anxiety. Similarly, in accordance with Verhaak et al. (2005), we did not find significant correlations between Problem Solving and perceived levels of State-Anxiety, neither in female nor in male infertile patients.

However, original and unexpected evidence for significant moderating effects of Problem Solving and Avoidant coping strategies were also found (H4).

In particular, moderation analyses showed that Problem Solving not only emerged as linked to increased levels of State-Anxiety (H3) but also played a negative moderating role, exacerbating in both partners the effects of Social Concern. This result supported the idea that the adoption of problem-management strategies could be inefficient and even counter-productive among infertile patients (Benyamini et al., 2008). We hypothesize this result could be interpreted by considering both the actual and perceived absence of control characterizing infertility condition, which may make ineffective all the efforts to re-establish it by actively rationalizing and making plans to handle frustration and reduce infertility-related social concerns.

Contrariwise, though the results from H3 indicate a significant association of Avoidant coping with increased levels of State-Anxiety, the recourse to these strategies revealed a significant moderating role in mitigating the negative effects of specific infertility-related stress dimensions, i.e., social concerns in male partners, need for parenthood in female partners, and couple’s relationship concerns in both members of infertile couples.

Therefore, these findings induced to hypothesize that also the recourse, to some extent, to avoidant strategies may potentially reduce perceived levels of anxiety. This by helping infertile couples to decrease the risk that infertility-related social concerns and couple’s relationship concerns become the center and need for parenthood becomes the main goal in their lives.

These findings give a further contribution to reinforcing the more recent branch of research, which sought to re-examine the role of avoidant strategies. It was, indeed, considered that one of its specific declinations, i.e., positive distraction, may not only foster a better adjustment but even promote well-being (Kleiber et al., 2002; Waugh et al., 2020). In this direction, the present study adds new acquisitions in the field of infertility research, suggesting that the adoption of avoidant and active-distractive strategies may effectively support both members of infertile couples in handling specific infertility-related stress dimensions.

Overall, findings endorsed the adoption of the transactional perspective to achieve a greater understanding of the role of coping strategies within the infertility-related stress process. Indeed, the study enlightened a specific and complex dynamic between individual characteristics and situational characteristics to be used for the assessment of both partners of infertile couples (Van den Broeck et al., 2010; Zurlo et al., 2020). It therefore follows several implications for clinical practice.

### Implications for Clinical Practice

Findings from the present study provided specific information on the pathways of associations between infertility-related stress dimensions, coping strategies, and anxiety in male and female partners of infertile couples, helping to develop patient-centered evidence-based counseling interventions.

First, considering that the relevant clinical levels of anxiety emerged in international research and were confirmed in the present study, our data suggested that structured programs should be developed to assure careful assessment, support, and monitoring of infertile patients’ perceived stress and psychological health.

In this direction, findings from the present study provided original evidence endorsing the adoption of a transactional perspective to achieve a greater understanding of the complex dynamics featuring infertility-related stress process and to develop tailored psychological interventions.

From this perspective, practitioners should carefully take into account the possibility that fostering coping strategies such as Seeking Social Support and Problem Solving, traditionally identified as adaptive and efficient to handle chronic stress, could be, instead, counter-productive to deal with infertility experience.

In the same direction, the findings from this study indicated that counseling interventions with infertile couples should consider the possibility to also promote the recourse, to some extent, to Avoidant coping strategies, in terms of positive distraction and seeking out individual and couple activities that may increase positive emotions in everyday life. This, indeed, can help infertile couples to protect themselves by distancing from infertility experience, which may, in some cases, entirely absorb their life. The recourse to avoidance can be helpful in patient-centered interventions with both partners of infertile couples perceiving intense couples’ relationship concerns. Moreover, its recourse can be helpful with male infertile patients suffering from social concerns, as well as with female infertile patients suffering from need for parenthood.

Nonetheless, data also fully supported the necessity to promote, within counseling interventions, the adoption of specific active coping strategies, such as Positive Attitude, considering also the necessity for practitioners to provide a meaningful space in which infertile patients could face, elaborate, signify, and re-elaborate their own experience.

### Limitations and Future Research

Despite these findings, some limitations of the study need to be addressed. First, one limitation is the cross-sectional design, and, therefore, no inferences about the temporal associations between predictors and outcomes can be suggested and no cause–effect relationships can be proposed. Despite this design having been considered useful to preliminarily test our hypotheses (Spector, 2019), future research could be developed with a longitudinal design. Second, self-report measures were used in the present study; hence, common method variance could not be ruled. Therefore, although common method variance does not necessarily influence the validity of findings (Fuller et al., 2016), future research could be developed including multi-source data. Third, in line with our objective, this research study re-examined the role of coping strategies in the associations between infertility-related stress dimensions and State-Anxiety on a general sample of male and female partners of infertile couples. Nevertheless, in future studies, it would be advisable to also explore further variables that could play a role in infertility-related stress process, such as socio-demographics (e.g., Age, Educational Level, Employment Status) and infertility-related parameters (Duration of Infertility, Type of Diagnosis, Previous Treatment). In addition, because of the inherently dyadic nature of infertility experience, future research could investigate infertility-related stress process by using a dyadic approach (e.g., by adopting the Actor–Partner Interdependence Model), also including measurement tools specifically designed to explore dyadic dimensions, such as the dyadic coping strategies (e.g., the Dyadic Coping Questionnaire; Bodenmann, 2000; Donato et al., 2009). Finally, although these findings could be of international interest, the study was carried out with a sample of Italian infertile couples. Therefore, future research could be developed with a cross-cultural design to test the generalizability of these results.

Despite the limitations reported previously, the study provided original and gender-specific evidence on the role of coping as moderators in the associations between infertility-related stress dimensions and psychological health. Findings can foster the development of more tailored evidence-based counseling interventions with infertile couples.

## Data Availability Statement

The raw data supporting the conclusions of this article will be made available by the authors, without undue reservation.

## Ethics Statement

The studies involving human participants were reviewed and approved by the Ethical Committee of Psychological Research of the University of Naples Federico II (IRB:34/2019). The patients/participants provided their written informed consent to participate in this study.

## Author Contributions

MCZ contributed to study conception and design, interpretation of data, drafting of article, and critical revision. MFCDV contributed to acquisition of data, analysis and interpretation of data, and drafting of article. FV contributed to analysis and interpretation of data and drafting of article. All authors read and approved the final article.

## Conflict of Interest

The authors declare that the research was conducted in the absence of any commercial or financial relationships that could be construed as a potential conflict of interest.
